# Sex differences in the acoustic structure of terrestrial alarm calls in vervet monkeys (*Chlorocebus pygerythrus*)

**DOI:** 10.1002/ajp.23674

**Published:** 2024-08-20

**Authors:** Colin Dubreuil, Hugh Notman, Louise Barrett, Peter Henzi, Mary Susan McDonald Pavelka

**Affiliations:** ^1^ Department of Anthropology and Archaeology University of Calgary Calgary Alberta Canada; ^2^ Faculty of Science and Engineering University of Wolverhampton Wolverhampton UK; ^3^ Department of Psychology University of Lethbridge Lethbridge Alberta Canada; ^4^ Department of Anthropology Athabasca University Athabasca Alberta Canada; ^5^ Applied Behavioural Ecology and Ecosystems Research Unit University of South Africa Gauteng South Africa

**Keywords:** alarm call, bioacoustics, call structure, *Chlorocebus pygerythrus*, communication, predator model, sexual selection

## Abstract

The alarm calls of vervet monkeys (*Chlorocebus pygerythrus*) have been the subject of considerable focus by researchers, owing primarily to the purported referential qualities of different alarm call types. With this focus on reference, acoustic variation among calls elicited by the same range of predators has typically been overlooked. Specifically, at least one type of alarm call—the terrestrial alarm—was described over 50 years ago as being acoustically distinct between males and females—a description that has largely eluded more systematic scrutiny. Here, we provide a quantitative acoustic analysis and comparison of terrestrial alarm calls produced by adult male and female vervet monkeys. We use a random forest model to determine which acoustic variables best distinguish between the calls of males and females, and use an unsupervised clustering technique to objectively determine whether alarms produced by each sex fall into discrete types. We found that the calls of males and females differed most in frequency‐based parameters, with male alarms containing more energy at lower frequencies relative to females. Calls produced by males were also of longer duration, and consisted of longer individual call elements relative to female calls. While calls generally fell into clusters associated with either male or female alarms, we found that some fell into atypical clusters given the caller's sex, and that the clusters themselves showed evidence of intergradation. We discuss these results in terms of potential differences in the function of, and motivation for, calling by males and females. We emphasize the need for a more holistic approach to the classification of vocal signals that considers contextual, functional, and structural variation.

## Research Highlights

- The calls of adult male vervet monkeys are acoustically distinguishable from those produced by adult females based on both spectral and temporal parameters.
- Acoustic intermediates and misassigned calls suggest sex differences in call structure are not solely attributable to sexual dimorphism.
- Differences suggest that barks produced by males may be under selection to advertize body size or competitive ability.

## Graphical abstract

What sets male and female vervet monkey alarm calls apart? Exploring sex differences in terrestrial alarm call structure.

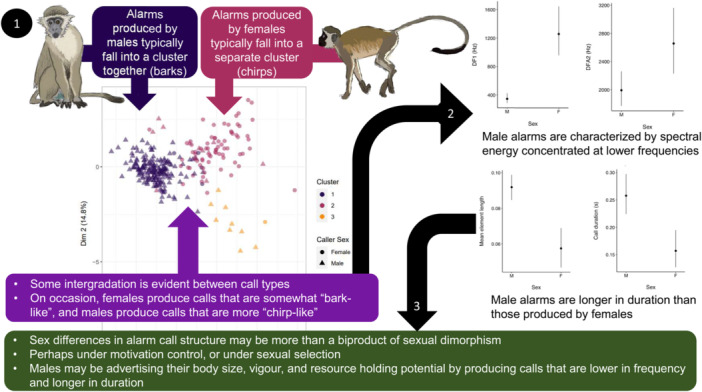

## 1 INTRODUCTION

Quantitative descriptions of the vocal repertoires of several primate species have revealed sex differences in the structure and production of vocal signals. These differences manifest as males and females producing specific vocalizations at different rates or in different contexts, or as differences in the acoustic structure of specific call types (e.g., Arnedo et al., 2010; Briseño‐Jaramillo et al., 2017; Dubreuil et al., 2015; Harris et al., 2006; Hohmann, 1991). In some species, particular calls are produced exclusively or more frequently by one sex (e.g., female copulation calls, Dixson, 1998; Pradhan et al., 2006; “loud” or “long” calls, Wich & Nunn, 2002; contact calls, Arnedo et al., 2010; Dubreuil et al., 2015; Lemasson et al., 2013). “Loud” or “long” calls for example tend to be produced more frequently by males and in a narrower range of contexts, even when females are physiologically capable of producing them (Briseño‐Jaramillo et al., 2017; Mitani & Stuht, 1998). Both male and female black and white colobus monkeys (*Colobus guereza*), for instance, produce loud “roar” vocalizations across a variety of contexts, but only males produce them during morning choruses (Harris et al., 2006). In several guenon species (genus *Cercopithecus*) loud calls are produced exclusively by males (Gautier & Gautier‐Hion, 1977; Zuberbühler, 2002) and can function to deter extra‐group males from approaching a caller's social group (e.g., *Cercopithecus mitis*; Fuller & Cords, 2017; Fuller, 2014). Because “loud” or “long” calls have been observed widely in a variety of mammalian taxa, and are often produced more frequently by males, they are hypothesized to play a role in territoriality and, by extension, in inter‐ and/or intra‐sexual selection (e.g., red deer, *Cervus elaphus*; Charlton et al., 2007; fin whales, *Balaenoptera physalus*; Croll et al., 2002: Chacma baboons, *Papio ursinus*: Kitchen et al., 2003a, 2003b, reviewed in Delgado, 2006; Mitani & Stuht, 1998; Snowdon, 2004).

The examples above of naming call types in species vocal repertoires highlight a persistent challenge in the study of animal acoustic communication, namely the inconsistent and subjective classification of vocalizations into often overlapping categories based on some combination of acoustic structure (i.e., what the call “sounds” like), context of usage, and/or putative function (Owren & Rendall, 2001; Seyfarth et al., 2010; Wadewitz et al., 2015). For example, some calls are sufficiently stereotyped and acoustically distinct from others, and can accordingly be defined categorically based primarily on structure (e.g., growls, barks, screams, “Pyow,” “Boom”; Bernstein et al., 2016; Fuller, 2014). Conversely, graded signals lack distinct acoustic boundaries, but may be distinguishable based on their association with particular contexts or arousal levels in callers (e.g., Fichtel & Hammerschmidt, 2002; Meise et al., 2011). The classification of calls into different descriptive ‘types’ is an expedient shorthand that nonetheless risks conflating the relationship between structural, contextual, and functional criteria. At the same time, a singular focus on any one of these classification methods (e.g., context or function) risks overlooking variation that exists along another dimension (e.g., acoustic structure).

The classification conundrum is perhaps best exemplified in the case of “alarm” calls, which is the (putative functional) term generally applied to vocalizations produced at the detection of a predator, and that typically elicit a range of antipredator responses in receivers including vigilance, mobbing, and evasive behaviors (Caro, 2005). Alarm vocalizations are often categorized both functionally and structurally (e.g., as “alarm barks,” Price et al., 2015). Although alarm calls vary considerably in structure both within and between species, they are often characterized by high amplitudes with their acoustic energy concentrated at frequencies in lower spectra. These characteristics are hypothesized to elicit alert responses in receivers, and facilitate distance propagation (Marten & Marler, 1977; Morton, 1975; Owren & Rendall, 2001). In this way, many alarm calls exhibit structural parallels with other long‐distance vocalizations, including calls that function in territoriality, group spacing (both inter and intra‐group), or mate defense and attraction, which are often referred to by the broad structural label “loud calls”; (e.g., Kitchen et al., 2015; Mitani & Stuht, 1998; Wich & Nunn, 2002). Moreover, in several species of primate, the term “loud call” has been applied to calls that function as alarms in at least some contexts (De Luna et al., 2010; Kitchen et al., 2003a, 2003b; Wich et al., 2003; Zuberbühler, 2001, 2009).

Although alarm calls have been central to research focusing on the mechanisms by which acoustic signals affect receiver behavior (Fischer, 2017; Townsend & Manser, 2013), there has been relatively less research investigating within‐species differences in the vocal repertoires of specific age and sex classes. However, sex differences in alarm calling behavior occurs in several primate species. For example, chacma baboon males produce “wahoo” vocalizations in response to predators, whereas females and juveniles produce “barks” (Fischer, Hammerschmidt, et al., 2001; Fischer, Metz, et al., 2001). Whereas the wahoo seems to serve a warning function for conspecifics, these calls are also associated with male competitive displays, and vary in structure based on caller rank, suggesting they may function in intra‐sexual selection (Fischer et al., 2004; Kitchen et al., 2003a, 2003b). Additionally, wahoo vocalizations contain cues to caller identity (Fischer et al., 2002), and thus may provide receivers with the ability to assess the competitive abilities of specific males (Delgado, 2006; Snowdon, 2004). Similarly, “pyows” produced by male blue monkeys (*Cercopithecus mitis*) appear to play a role in predator avoidance, group cohesion, and mate defense/attraction, based on their use across several contexts, and the responses they elicit from receivers (Fuller & Cords, 2017).

For over 50 years, vervet monkeys (*Chlorocebus pygerythrus*) have been a central focus for studying alarm behavior in nonhuman primates (Struhsaker, 1967; Seyfarth et al., 1980a, 1980b). Like many guenons, vervet monkeys produce multiple acoustically distinct alarm calls (Price et al., 2015; Struhsaker, 1967; Seyfarth et al., 1980a, 1980b). The original descriptions by Struhsaker (1967) identified three primary call classes based on the predator type that elicited them (context), that were further delineated into four call types based on acoustic structure. These were snake alarms (“chutter”), aerial alarms (“rraup”), and two distinct call types that were both produced in response to mammalian predators—“chirps” and “threat alarm barks”.

According to Struhsaker's original descriptions of vervet alarms (Struhsaker, 1967), adult females and juveniles of both sexes produce “chirps” in response to mammalian predators, whereas adult and subadult males produce “threat alarm barks.” Both call types are described as low‐frequency, high‐amplitude calls, although threat alarm barks consist of multiple units comprising a series of ex‐ and inhalations, whereas chirps consist only of exhalations and are “abrupt,” “short,” and “sharp sounding” (Struhsaker, 1967). Because these calls are elicited in response to similar predator classes and elicit similar responses from receivers, they are often regarded as functionally equivalent (Seyfarth et al., 1980a, 1980b, Price et al., 2015). This may reflect a long‐held tendency to regard vervet monkey alarm calls as an example of “functionally referential” communication (Townsend & Manser, 2013), generating its own legacy of research focused on the putative informational content of these signals (Owren & Bernacki, 1988, Rendall, 2021, Seyfarth & Cheney, 1990; Seyfarth et al., 1980a, 1980b). Under the referential model, the perceptually salient sex differences in acoustic structure of alarms raise the curious implication that two distinct vocal types have either converged on the same referent (i.e., terrestrial predators) in a manner analogous to synonymy in human language. Conversely, alarm calls might have experienced acoustic divergence that reflects different direct or indirect functions of alarm calling in each sex, such as advertising male quality.

More recent studies have shown that the male's alarm bark is frequently produced in contexts unrelated to predation (Price et al., 2015), and that responses to alarms vary among populations (Ducheminsky et al., 2014). Male vervets, for example, produce calls that are acoustically similar to the “threat alarm bark” in male‐male antagonistic contexts as well as in response to predators (Price et al., 2015), and receiver responses vary according to factors such as group size, group dispersal, habitat structure, eliciting context, and relative predation risk (Deshpande et al., 2023; Dubreuil et al., 2023; Ducheminsky et al., 2014; Enstam & Isbell, 2002; Mohr et al., 2023). These findings suggest a more general link between the production of these signals and the callers' affective state in response to threatening/aversive situations, requiring receivers to make use of a broader range of ecological variables and cues to determine the best response (Wheeler & Fischer, 2012). Accordingly, sex differences in alarm call structure may represent an additional dimension of contextual salience that influences receiver responses and subsequent behaviors.

Although described qualitatively (Seyfarth et al., 1980b; Struhsaker, 1967), the acoustic differences that characterize the alarm calls of vervet males and females have yet to be quantified systematically. Commonly, vocal signals have been classified by observers subjectively by ear, in combination with visual examinations of spectrograms and power spectra (see Fischer et al., 2017 for review). These methods, in conjunction with observational data surrounding the contexts in which different calls are produced, have been used to separate a species' vocalizations into functional units (call types). Current techniques often still focus on the use of spectrograms for signal classification but benefit from the ability to use automated feature extraction, thus reducing observer biases when measuring the actual acoustic variables (Fischer et al., 2013; Fischer et al., 2017). It is also possible to use unsupervised clustering methods to group signals based on their acoustic parameters alone, without regard to the contexts in which they were produced (Fischer et al., 2013; Wadewitz et al., 2015). An advantage of this approach is a reduction in the subjectivity of classifying call types by ear. Moreover, clustering quantifies the distance between individual calls or broader call types in acoustic space, and can reveal calls that exist as acoustic intermediates between more stereotyped categories (e.g., Hammerschmidt & Fischer, 1998; Tallet et al., 2013; Wadewitz et al., 2015).

Here, we provide a quantitative analysis of the vervet monkey's terrestrial alarm calls, with a focus on assessing objectively the sex differences that characterize their acoustic structure. We also explore how consistent the relationship is between call structure and caller sex. We use a statistical clustering technique in combination with a random forest model and a series of linear models to identify the acoustic variables that best distinguish between the alarm calls of males and females, and to determine the extent to which the acoustic structure of male and female calls overlap. By quantifying sex differences in the terrestrial alarm calls of vervet monkeys we hope to spur further considerations of the functional significance of this variation, the relationship between the form and (potentially) sex specific functions of these signals, and how such variation might relate to putative referential mechanisms that have been described extensively elsewhere for this species.

## 2 METHODS

### 2.1 Ethics statement

This research adheres to the ASAB/ABS Guidelines for the use of animals in research. The procedures presented here were approved by the University of Lethbridge Animal Welfare Committee (Protocols 0702 and 1505).

### 2.2 Study site and population

We collected data for this study at the Samara Game Reserve, Eastern Cape Province, South Africa (32° 22′S, 24°52′E), from May 2016 to May 2017. Author CD and several trained research assistants collected data from three habituated groups of vervet monkeys, two of which have been habituated to the presence of researchers since 2008, and the third since 2012. All three study groups occupy adjacent territories in semi‐arid riverine *Acacia* woodland (Pasternak et al., 2013). We have recorded all births, deaths, and migration events since research on this population began. The number of adult males and females per group varied over the course of data collection for the current study (adult males range 4–10, adult females range 6–13, Table 1).

**Table 1:** Number of adult males and adult females across the three study groups over the course of data collection.

Social Group	Males	Females
1 (PT)	6–9	8–9
2 (RBM)	4–5	6–10
3 (RST)	8–10	9–13

*Note*: Counts are represented as a range. Variation in values are due to deaths, immigrations, and emigrations throughout the study period.

### 2.3 Vocal data collection

We collected vocal recordings and contextual data surrounding the production of calls during 10‐h follows of the three study groups, 5 days a week. In the winter, the daily 10‐h follow period coincided with daylight hours, meaning we followed the subjects from when they first left their sleeping trees until they returned to their sleeping site at the end of the day. In the summer months, we balanced daily group follows between (a) days that started at sunrise and ended 10 h later, and (b) days that ended at sundown, having started 10 h earlier. Each day, author CD and one trained field assistant followed one of the three study groups, equipped with a Sennheiser ME67 directional condenser microphone, and a Marantz PMD661 digital field recorder. We made recordings at a sampling rate of 48 kHz, and a bit rate of 1536 kbps. To reduce wind and handling noise, we equipped the microphone with a blimp windshield and shock mounting system with a pistol grip (Sennheiser MZS20‐1 Combo Mount/Grip/Stand). We rotated between which of the three study groups were followed with the microphone to balance our recordings across social groups over the study period. We made recordings of alarm calls in two conditions—(1) opportunistically when alarm calls occurred naturally at the detection of a predator or (2) during experimental predator model presentations. Although vervet monkeys have been described producing bark‐like calls during non‐predatory contexts (e.g., during male‐male aggression or intergroup encounters; personal observation, Price et al., 2015), our sample consisted entirely of calls produced in predatory contexts. This limitation is due in part to the difficulty of recording “aggression barks,” which often occurred quickly, in bouts of 2 or 3 calls that were usually over before we were able to locate the caller for recording. When alarm calls occurred under natural predator encounter conditions, either CD or a trained field assistant identified the eliciting stimulus. We would then locate a calling animal and record its vocalizations while dictating its identity into a headset microphone. Whenever possible, we would attempt to record more than one individual per alarm bout.

We supplemented our naturalistic audio recordings by conducting experimental predator model presentations. We presented predator models at intervals of no fewer than 14 days apart to any given group to avoid habituating the subjects to the mounts. In preparation for a predator model presentation, we would place a stuffed caracal (*Caracal caracal*) concealed under a blanket (gray, brown, or forest green in color) in the path of a target group as it traveled, out of view of any group members. Pilot work with our study groups revealed that the vervet monkey's response to the predator model resembled those of their response to naturalistic encounters with terrestrial predators. When we were confident that no monkeys could see the covered mount, we would remove the cover and move away in preparation to record any vocalizations produced by the monkeys. Once the mount had been spotted, we would attempt to record as many different individuals calling as possible. This number varied a great deal between trials based on the number of individuals calling, as well as the position of individuals in trees; during both the predator model presentations and natural predator encounters, subjects, particularly younger individuals and females, would move higher into trees before/while calling, where they would stay until the predator had moved away, or the predator model had been removed. Individuals became harder to identify in these conditions, and as a result, adult females were considerably more difficult to record than adult males, who often moved lower in the trees as they called and approached the eliciting predator/predator model. The distance between the microphone and the callers during both naturally occurring and experimentally induced alarm bouts was between 3 and 10 m.

### 2.4 Data analysis

#### 2.4.1 Selecting calls for analysis

We selected calls that were both clear of background noise and had a high signal‐to‐noise ratio for acoustic analysis. In both naturally occurring as well as experimentally‐induced bouts of alarm calling, a single individual would generally produce a large number of calls. Calls produced by a single individual could consist of a different number of elements, defined as a continuous tracing on an oscillogram whose energy was above that of the ambient background noise. Additionally, some calls produced by a given individual also contained audible exhale units, whereas others did not (Price et al., 2015, Figure 1). Because of this variation within a given alarm bout, we selected and analyzed multiple calls from the same bout for each individual, as no one call was fully representative of all the calls produced in that bout. In total, we selected 79 alarm calls produced by nine individual adult females (range 1–19 calls per individual, median = 6), and 207 calls produced by 17 individual adult males (range 3–31 calls per individual, median = 8). Of these, 237 calls were recorded during experimental predator mount presentations (177 from males, and 60 from females), and 49 were taken from natural predator encounters (30 from males, and 19 from females; Table 2). The number of individual calls taken from a single calling event (a single predator encounter or predator model presentation) ranged from 1 to 31. The maximum number of calls taken from any one individual during a single bout was 15. The modal number of calls taken from a single individual during a single bout was 4 (mean 5).

**Figure 1:**
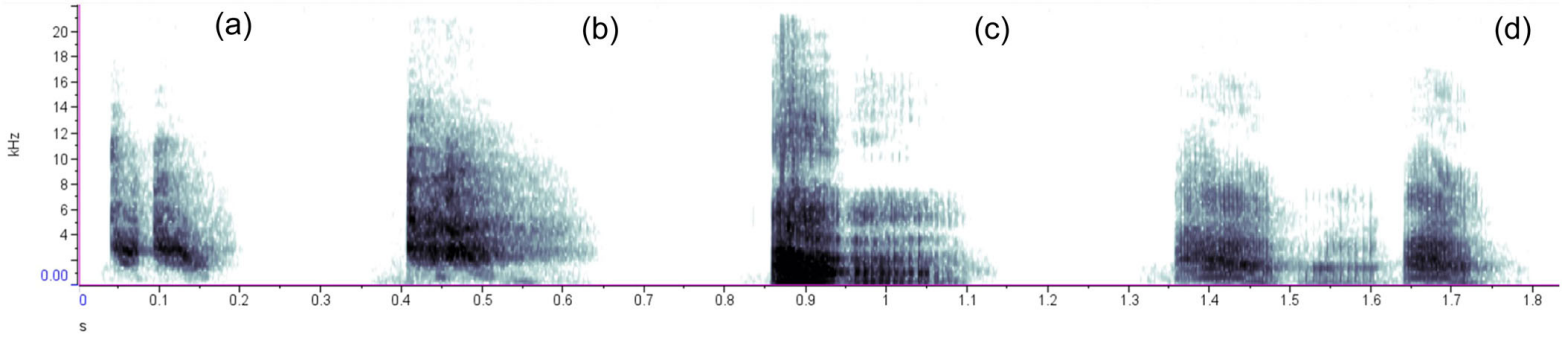
Spectrograms illustrating typical exemplars of chirps and threat alarm barks: (a) chirp produced by an adult female consisting of two separate call elements; (b) chirp produced by an adult female consisting of a single call element; (c) threat alarm bark produced by an adult male consisting of two elements. The second element is an audible inhaled element. (d) Threat alarm bark produced by an adult male consisting of three elements. The second (middle) element is an audible inhaled element. Calls shown here are produced by four different individuals during four separate alarm bouts and have been organized into this sequence for illustrative purposes only. Spectrograms were made with a 163 FFT and a Hann window (Raven Pro version 1.6, https://ravensoundsoftware.com/).

**Table 2:** Number of calls analyzed from both naturalistic predator encounters and from experimental predator mounts for both females and males.

Sex	No. of calls			No. individuals	Median no. calls per individual (range)
	Total	Naturalistic encounters	Predator mounts		
Female	79	60	19	9	6 (1–19)
Male	207	177	30	17	8 (3–31)
Total	286	237	49	26	7.5 (1–31)

*Note*: The number of individual female and male callers in the sample, alongside the median and range of calls taken per individual, are also presented.

#### 2.4.2 Acoustic analysis

Before analysis, we high‐pass filtered calls at 100 hertz (Hz) to remove any noise below the lowest frequency of the call (Raven Pro, v1.5). We labeled each call element and padded them with 0.2‐s silent margins before resampled them to 16 kHz using Avisoft SASLab Pro. We performed a Fast Fourier transform (Hamming window function, 1024 points, 93.75% overlap) to generate a spectrogram for each call element. We exported spectrograms and analyzed them using a custom sound analysis software developed by K. Hammerschmidt (LMA—Lautmusteranalyse v. 2018_0.4).

Many of the calls we recorded lacked a clear harmonic structure (i.e., they were aperiodic), making measurements of fundamental frequency impossible in many instances. Instead, we followed the approach outlined in Price et al., (2015) (see also Fischer et al., 2013), and measured variables relating to the broader distribution of energy within the calls (Table 3).

**Table 3:** Acoustic parameters measured from calls.

Temporal measurements—Based on entire call	
Call Duration*	**Full duration of the call**
Number of elements*	**Number of call elements within a call**
Mean element duration*	Mean element duration within a call
Spectral measurements—Based on single elements	
Pfmean	Mean peak frequency (PF)[Hz]
Pfmaloc*	Location of the maximum peak frequency [(1/duration)*location]
Pfmiloc*	location of the minimum PF [(1/duration)*location]
Pfjump*	**maximum difference between successive PF's [Hz]**
**Pftrfak**	**factor of linear trend of PF (global modulation: −1 to 1)**
Pftrmean	Mean deviation between PF and linear trend [Hz]
Pftrmax	Maximum deviation between PF and linear trend [Hz]
DFA1	Distribution of the frequency amplitudes 1: Mean first quartile of amplitude in spectrum
DFA2*	**Distribution of the frequency amplitudes 2: Mean second quartile of amplitude in spectrum**
DFA3	Distribution of the frequency amplitudes 3: Mean third quartile of amplitude in spectrum
**mean_noise**	**Mean value of noise within call. 0 = pure tone, 1 = random noise**
DF1*	**Mean frequency of 1st dominant frequency [Hz]**
**df1maxloc**	Location of the maximum frequency 1st DF [(1/duration)*location]
**df1minloc**	Location of the minimum frequency 1st DF [(1/duration)*location]
**df1trfac**	**Factor of linear trend of 1st DF (global modulation)**

*Note*: Acoustic parameters used in random forest models are highlighted with an asterisk (*). Variables that were entered into the cluster analysis are indicated in bold.

Abbreviation: Hz, hertz.

#### 2.4.3 Variable selection

We used a two‐step process to reduce the number of acoustic parameters in our sex differences analyses (see random forest analysis below). First, we conducted a principal components analysis to reduce our 18 acoustic variables to a set of uncorrelated principal components. We identified five principal components (PC) that together accounted for 75% of the variance in the data. The first PC correlated strongly with variables that describe the energy distribution at different frequencies within the calls (DFA 1, 2, 3, and peak frequency). The second PC correlated with changes/movement of the peak frequency throughout the calls (pfjump, pftrmax, pftrmean). PC 3 correlated strongly with the number of elements in the calls, and call duration. PCs 4 and 5 correlated most strongly with the location of the minimum and maximum peak frequencies in the calls respectively (pfmiloc and pfmaloc) and captured variance in parameters related to the relative position and the movement of the dominant and peak frequency bands throughout the calls' duration (PC4: pfmiloc, df1maxloc, df1trfac; PC5: pfmaloc). We selected the five acoustic parameters that loaded most strongly onto each of the principal components (one parameter per component) to represent each of those dimensions (viz. PC 1: DFA2, PC 2: pfjump, PC 3: number of call elements, PC4: pfmiloc, and PC 5: pfmaloc). We also selected three additional acoustic variables that, based on the source filter framework (Fitch & Hauser, 2003), were likely to vary with caller size: call duration, average element duration, and frequency of the first dominant frequency band (DF1).

#### 2.4.4 Statistical analysis

##### 2.4.4.1 Random forest models

We used a random forest model (Breiman, 2001) to, (a) assess how well terrestrial alarm calls could be distinguished by sex of the caller, and (b) to determine which acoustic variables were most important for making this distinction. Random forest models are a machine learning technique that extend standard classification and regression tree (CART) analysis (Kassambara, 2018). The random forest model classifies each case in the sample to either the correct group (correct assignment), or to an incorrect group (incorrect assignment). Models were “trained” using a randomly selected 70% of the data (training data set). The remaining 30% of the data were used as the “validation data set” to determine whether the random forest models generalize well to data that were not used to train the model (Kassambara, 2018). We entered our eight representative acoustic variables into the model as independent variables, and caller sex as the dependent (outcome) variable. We ran the model using the “caret” (Classification and regression training) package (Kuhn et al., 2023) in R4.2.2 (R‐Core‐Team, 2022).

Because the results of classification problems can be affected by imbalanced data, we re‐ran the random forest model, applying the Synthetic Minority Oversampling Technique (SMOTE) algorithm to our minority class (Chawla et al., 2002). SMOTE generates synthetic data points by randomly selecting cases from the minority class (in this case, female calls), and finding the *k* nearest neighbors to that data point within the feature space. The algorithm creates synthetic cases between the original point and a randomly selected nearest neighbor, repeating these steps on different cases until a desired number of synthetic samples are generated to create a balanced data set. Results using the balanced data set were comparable to those of the analysis based on the original sample. Therefore, for ease of interpretation, we present the results of the original, imbalanced model in the main text, and present results of the balanced model as Supporting Information S1.

We used the “mean decrease in Gini” and “mean decrease in Accuracy” indices to evaluate the relative “importance” (Breiman, 2001) of the different acoustic variables in predicting caller sex. The “mean decrease in Gini” index is a measure of how much the purity of the nodes of the decision trees generated by the model decrease if a given variable was randomly permuted with regard to the outcome variable. Mean decrease in accuracy describes the proportion of calls that would be misclassified in terms of the outcome variable if a given independent variable were randomly permuted. Variables with higher Gini and accuracy index values are considered to be more important for the given classification problem. We assessed variable importance for the random forest models task using the importance() function from the randomForest package (Liaw & Wiener, 2002).

#### 2.4.5 Sex differences in representative acoustic variables

We ran a set of eight generalized linear mixed models to determine whether caller sex had an effect on any of the eight representative acoustic variables. For each model, we specified one of the acoustic variables as the response variable, and caller sex (male/female) as the predictor. Individual caller identity was entered as a random effect to account for repeated sampling. Model families and link functions for each of the eight analyses are listed in Supporting Information S2: Tables S1–S8. We ran the models in a Bayesian framework, using the “brms” package (Bürkner, 2017). We specified weakly informative priors (normal (0, 1)) and ran models with four chains and 3000 iterations (warmup = 1000; thin = 1). Chain convergence was confirmed ($\hat{R}$ ≤ 1.01) for all models and effective sample sizes were satisfactory. The “pp_check” function from the “bayesplot” package (Gabry & Mahr, 2022; Gabry et al., 2019) was used to allocate and evaluate the performance of the specified distributions and link functions in fitting the model. We considered the uncertainty in sign and magnitude of posterior distributions when interpreting model outcomes (Gelman & Carlin, 2014). We set the credible intervals at 95% because of their interpretive familiarity and used these, backed by “probability of direction” estimates (pd) and *R* ^2^ values from the “bayestest” package (Makowski et al., 2019), to evaluate model outcomes.

##### 2.4.5.1 Cluster analysis

We performed a hierarchical, agglomerative cluster analysis to investigate whether the calls of males and females fall into objectively distinct categories, corresponding to the “chirps” and “threat alarm barks” as described by Struhsaker (1967). Agglomerative clustering is a type of hierarchical cluster analysis whereby each observation is initially treated as its own cluster. At each iteration, pairs of clusters that are the most similar are joined with one another to create larger and larger clusters. This process is repeated until all observations are joined together into a single large cluster. Hierarchical clustering requires the user to set the number of desired clusters before running the analysis. Although previous work on this species suggests that a two‐cluster solution would be optimal (a separate cluster for “chirps” and “threat alarm barks,” respectively), we wanted to let the data dictate the optimum number of clusters, allowing for an objective clustering solution based solely on the actual data. To this end, we used the NbClust () function from the NbClust package, (Charrad et al., 2014) to suggest an optimal number of clusters based on a total of 26 indices. We used the eclust() function from the factoextra package for our cluster analysis. We used the “hclust” clustering function, and calculated dissimilarities between observations using Euclidean distance measures. We set the agglomeration method to “ward.D2”. Based on the results provided by the NbClust() function, we set the number of clusters at three (*k* = 3).

Because cluster analyses can be skewed by highly correlated variables (Kassambara, 2017), we used a set of 12 uncorrelated acoustic variables (out of our initial 18) for this analysis. When two or more of our original 18 variables were highly correlated (*r* > 0.75), we retained the variable that was rated highest in terms of its importance for discriminating between the calls of males and females by the random forest model. Importantly, although we aimed to keep a selection of the most “important” variables for distinguishing between males and females from the random forest models, we also retained variables that were rated as being less important to avoid pre‐biasing the cluster analyses into identifying clusters that corresponded to sex differences in alarm structure. The variables included in the cluster analysis are highlighted in Table 1. All variables were standardized for the cluster analysis (Kassambara, 2017).

We evaluated our cluster solution using the silhouette method. Silhouette values are a commonly used method of evaluating cluster solutions that take into account how close a case (call) is to the center of its own cluster, and how far away it is from the center of any other neighboring cluster(s). Each case is given a value from −1 to 1, with higher values representing well‐clustered cases characterized by small within‐cluster distance and high between‐cluster distance (Kassambara, 2017; Kaufman & Rousseeuw, 2009). Cases with silhouette values close to zero suggest a call was relatively distant from the center of its own cluster. Negative silhouette values represent poorly clustered cases. The average silhouette value for the entire cluster solution can be used as a metric to evaluate how discrete the clusters are from one another. Cluster solutions with average silhouette values of 0.51 and above are generally considered to represent cases where data can be partitioned into reasonably discrete clusters (Kaufman & Rousseeuw, 2009). Values lower than 0.51 suggest intergradation between clusters.

To assess whether caller sex predicted cluster membership while controlling for caller ID, we ran a Generalized Linear Mixed Model (GLMM) specifying cluster membership as the dependent variable, and caller sex (Male/Female) as the predictor. We entered caller identity as a random effect to account for repeated sampling. We specified the categorical family with a logit link. We followed the same analytical and interpretive procedures for the generalized linear mixed models described above (see section—Sex differences in representative acoustic variables). Note that *R* ^2^ values cannot currently be estimated for this model family, so model outcomes were evaluated solely based on 95% credible intervals and probability of direction (PD) estimates.

## 3 RESULTS

### 3.1 Random forest model

The model was able to distinguish between the calls of males and females with high accuracy (97.01% accuracy, OOB error = 2.99%), and had a sensitivity and specificity of 98.39% and 91.30% respectively. Classification error was low for calls by males (0.01) and calls by females (0.07). The model accurately predicted caller sex in 96.47% of the samples from the validation data set (95% CIs: 90.03, 99.27), which was better than chance expectation (permutation test; *p* < 0.001, expected classification accuracy by chance = ~61%). Calls were classified correctly by sex at levels that exceeded chance (Adult males: 96.82% accuracy vs ~73% accuracy expected by chance, *p* < 0.001; Adult females: 95.65% accuracy vs. ~27% accuracy expected by chance, *p* < 0.001).

Both variable importance measures indicated the same relative importance of the eight representative acoustic variables (Table 4). Parameters related to the frequency content of the calls (DF1 and DFA2) were rated as the most important variables for discriminating between the calls of males and females, followed by the duration of the individual elements that made up the calls, and the duration of the calls themselves. These variables were followed by parameters related to the relative position of the maximum peak frequency within the calls (pfmaloc), the movement of the peak frequency (pfjump), and the position of the minimum peak frequency (pfmiloc). The number of elements in the call was rated as the least important variable for distinguishing between the calls of males and females.

**Table 4:** Variable importance (mean decrease in Accuracy and Gini) for the random forest model.

Acoustic variable	Mean decrease in accuracy	Mean decrease Gini
**DF1**	**35.29**	**30.84**
**DFA2**	**22.29**	**23.43**
**Mean element duration**	**18.94**	**11.60**
Call duration	7.11	4.50
pfmaloc	6.71	4.09
pfjump	5.44	3.28
pfmiloc	5.39	1.91
Number of elements	4.90	0.99
Mean variable importance	13.26	10.08

*Note*: Bolded variables and values represent cases where the calculated variable importance index was higher than the average variable importance for all eight acoustic parameters.

**Figure 2:**
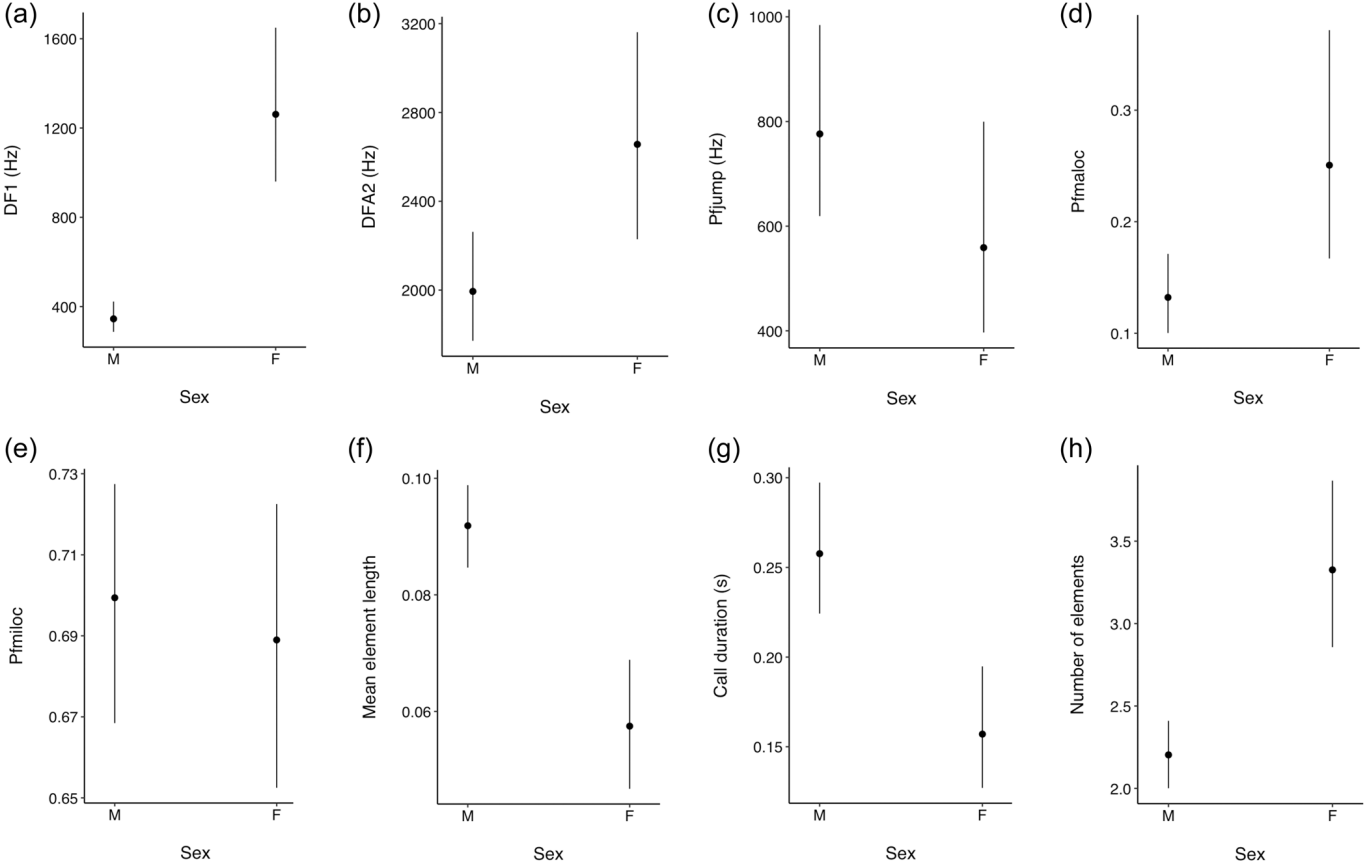
Predictive marginal means (±95% credible interval) for the relationship between sex (M: male; F: female) and (a) DF1, (b) DFA2, (c) Pfjump, (d) Pfmaloc, (e) Pfmiloc, (f) Mean element duration, (g) Call duration, and (h) Number of elements.

The GLMM models used to address sex differences in the representative acoustic variables identified meaningful sex differences for six of the eight variables (Table 5; Figure 2). Of these, DF1, and mean element duration showed the most marked sex differences, with sex explaining 56% and 29% of the variance in these variables respectively. Sex differences in DFA2 were smaller, but quite precise (i.e., the credible intervals for the estimate were narrow—see Table 5), with 26% of the variance in DFA2 being explained by sex (Table 5). Although sex was a reasonable discriminator for both pfmaloc and call duration, the amount of explained variance was very much lower in each case. Finally, the outcome for number of elements (with call duration as an exposure variable), despite the relative precision and magnitude of the difference, should be treated with caution as the posterior predictions generated by the model were very imprecise (see Supporting Information S2 for full model results).

**Table 5:** Posterior density estimates of the effects of sex (ref: female) on alarm call parameters.

Variable	®Model ± S.E.	Lower 95% CI	Upper 95% CI	R2Marginal	R2Conditional	PD (%)
**DF1**	**7.14** ± **0.14**	**−1.69**	**−0.98**	**0.56**	**0.90**	**100.00**
**DFA2**	**−0.29** ± **0.11**	**−0.50**	**−0.007**	**0.25**	**0.72**	**99.36**
Pfjump	6.33 ± 0.17	−0.08	0.74	0.02	0.18	94.40
**Pfmaloc**	**−0.38** ± **0.20**	**−1.11**	**−1.19**	**0.06**	**0.12**	**99.61**
Pfmiloc	0.69 ± 0.02	−0.02	0.04	0.001	0.002	74.58
**MEA**	**0.06** ± **0.01**	**0.02**	**0.05**	**0.29**	**0.44**	**99.98**
**CD**	**−1.85** ± **0.11**	**0.24**	**0.75**	**0.07**	**0.15**	**99.95**
**NOE**	**2.67** ± **0.08**	**−0.59**	**−0.23**	**0.84**	**0.85**	**100.00**

*Note*: Variables for which we identified meaningful sex differences are presented in bold. β, slope of the predictor; CD, call duration; CI, credible interval; MEA, mean element duration; NOE, number of elements; PD, probability of direction; S.E., standard error of the intercept.

### 3.2 Cluster analysis

**Figure 3:**
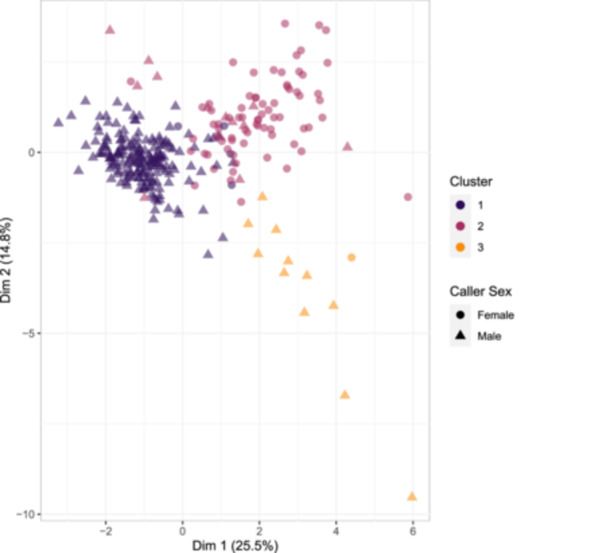
Vocalizations plotted against two principal components derived from the 12 acoustic parameters entered into the cluster analysis. Individual calls are color‐coded based on their assigned cluster membership. The shapes of the points (Triangles and Circles) represent calls produced by males and females respectively. Cluster one contains 188 calls produced by 17 individual males and three females. Cluster 2 contains 86 calls produced by nine individual females and six males. Cluster 3 contains 12 calls produced by a single individual male and a single female.

The three‐cluster solution resulted in clusters containing 188, 86, and 12 calls respectively (Table 6, Figure 3). Although all three clusters contained calls produced by both males and females, 88.4% of calls produced by adult males fell into the first cluster. Conversely, 92% of calls produced by adult females fell into the second cluster. Of the 188 calls in the first cluster, 183 (97.3%) were produced by males, whereas 73 of the 86 calls in cluster 2 (~85%) were produced by females. This sex bias in cluster membership was confirmed by the results of the GLMM that, after controlling for caller ID, indicated that calls produced by males were more likely to be grouped into cluster 1 relative to cluster 2 (Estimate = 2.40, Lower 95% CI: 1.13; Upper 95% CI: 3.57; pd: 99.9%—reference variables: Cluster 2, Males), and that calls produced by females were more likely to be grouped into cluster 2 relative to cluster 1 (Estimate = −3.31, Lower 95% CI: −4.95; Upper 95% CI: −1.06; pd: 99.7%). Full model outcomes are available in Supporting Information S2: Table S9). Cluster 3 was also biassed in terms of sex, with 92% of the 12 calls in that cluster being produced by a single male ‐ the remaining call being produced by a female. Importantly, the individual male who contributed calls to cluster three contributed 52% (*n* = 16) of his calls to cluster 1, 15% (*n* = 4) of his calls to cluster 2, and 35% (*n* = 11) calls to cluster 3. Similarly, the female who contributed to cluster 3 contributed 94% (*n* = 17) of her calls to cluster 2, and only 6% (*n* = 1) call to cluster 3. Not surprisingly, after controlling for caller ID, we detected no meaningful relationship between sex and membership in cluster 3 (Estimate = −0.49, Lower 95% CI: −2.29; Upper 95% CI: 1.35; pd: 70.4%. Reference variables: Cluster 2, Males).

**Table 6:** Cluster composition showing the number of calls grouped into each cluster, the number of calls from each sex that fell into each cluster, and the number of individuals whose calls were placed into each cluster.

Cluster	No. calls (% of all calls)	No. of contributing calls per sex (% of cluster, % of calls from each sex)		No. of contributing individuals by sex (% of cluster, % of individuals of each sex)		
		Female	Male	Females		Males
1	188 (66%)	5 (3%, 6%)	183 (97%, 88%)	3 (15%, 33%)		17 (85%, 100%)
2	86 (30%)	73 (85%, 92%)	13 (15%, 6%)	9 (60%, 100%)		6 (40%, 35%)
3	12 (4%)	1 (8%, 1%)	11 (92%, 5%)	1 (50%, 11%)		1 (50%, 6%)
Total	286	79	207	9		17

The five female calls that fell into cluster 1 (the predominantly male cluster) were produced by a total of three individual females, all of which had most of their calls assigned to the predominantly female cluster (cluster 2). The 13 calls produced by males that fell into cluster 2 were produced by six individual males. Again, each of these males contributed more calls that were clustered into cluster 1 (the predominantly male cluster) than they did to cluster 2 (Supporting Information S2: Table S10).

The mean silhouette value for the entire cluster solution was 0.27. Cluster 1 (the larger, primarily male cluster) had a silhouette value of 0.39, whereas the second (primarily female) cluster and third clusters had silhouette values of 0.02 and 0.20, respectively, thus suggesting that relative to cluster 1, the calls in clusters 2 and 3 were less stereotyped in structure. Still, the silhouette value of 0.39 suggests only weakly structured data (Kaufman & Rousseeuw, 2009).

## 4 DISCUSSION

Our results demonstrate that male and female vervet monkeys produce acoustically distinct calls in response to the same eliciting context—i.e., at the detection of a terrestrial predator. Our acoustic analyses build on qualitative descriptions of sex‐specific alarm call variants previously reported in this species. Specifically, our results show that male and female alarm calls differ primarily in the overall energy distribution throughout the call, with males producing alarms with lower first dominant frequency bands (DF1), and lower overall energy distribution (DFA2) relative to those produced by females. Additionally, the calls of males and females differed significantly in terms of call duration and in the duration of the individual elements that constitute each call. Males generally produced calls that were longer than those produced by females, and whose individual elements were longer than females as well.

The specific sex differences in acoustic properties reported here are consistent with the predictions of source‐filter theory (Morton, 1977; Taylor & Reby, 2010), in which several acoustic variables are likely to be indexical of body size in mammals, including fundamental frequency (associated with the size of the vocal folds); formant frequencies (associated with the dimensions of vocal tract) and call duration (associated with lung volume). Although we did not measure fundamental frequency or formant frequencies here, acoustic measures related to the overall distribution of acoustic energy, including the lowest dominant frequency band (DF1), and the broad distribution of energy throughout the spectrum (Distribution of the frequency amplitudes; e.g., DFA 2), are predicted to correlate with these two variables. Adult vervet monkeys exhibit body size sexual dimorphism, in which males are larger, and weigh more than females (Turner et al., 1997—females average 3.3 kg, and males 5.5 kg at Samara; Pasternak et al., 2013). As such, a plausible explanation for the acoustic differences between male and female vervet alarm calls is that they reflect differences in the underlying vocal physiologies and body sizes of males and females.

Although sex differences in body size might explain some structural differences in alarm calls, body size dimorphism does not readily explain why 6 of the 17 adult males in our sample did, on occasion produce vocalizations at predator mounts that grouped more strongly into the female cluster (meaning they produced calls that were more acoustically similar to a stereotypical “chirp,” generally produced by females), or why 3 of the 9 females produced calls clustered more closely with the more typically “male” cluster. One possibility is that the soft tissues involved in vocal production (which are less constrained by body size) vary continuously in a manner that mirrors the observed continuum in acoustic structure observed here. This being the case, some individuals may produce alarms that typically fall into the graded acoustic space between the more typical “chirp” and “threat alarm bark.” Future studies should explore soft classification approaches (e.g., fuzzy clustering) which better allow calls to be identified as intermediates between clusters (Wadewitz et al., 2015). Regardless, all individuals that produced calls that were atypical for their sex contributed a greater proportion of calls to their own sex's representative cluster. Crucially, larger sample sizes would be required to draw conclusions regarding how commonly males and females produce calls that are atypical of their own sex, and whether some individuals are more or less likely to do so. Another possibility is that, as with the classically described “loud” or “territorial” calls in species that produce them, adults of both sexes are *capable* of emitting alarms that are structurally more typical of the opposite sex (i.e., they are not physiologically constrained from producing either), but do so only occasionally and, perhaps, in specific contexts. This situation has been observed in the loud calling of a number of species in which males overwhelmingly produce them relative to females, such as chimpanzee “pant hoots” (Marler & Tenaza, 1977; Clark‐Arcadi, 1996; Notman & Rendall, 2005), and occurs even in species where males possess anatomical specializations for loud call production that are lacking in females (e.g., Black howlers, *Allouata pigra*; Briseño‐Jaramillo et al., 2017; Van Belle, 2015). In these instances, the male‐typical loud call has been reported in the female vocal repertoire, but they produce them much less frequently.

If males and females can produce similar‐sounding calls in response to the same eliciting stimulus (even if they do not frequently do so) despite differences in body size, it is possible that selection is favoring different usage patterns that are independent of physiological constraints imposed by size differences. This phenomenon has been observed in cases of vocal mimicry, particularly in the juveniles of mammalian species that experience high juvenile predation, and which thus benefit from mimicking the alarm calls of adults, despite differences in body size between adults and juveniles (e.g., Sciurids; Matrosova et al., 2007; Swan & Hare, 2008; Volodina et al., 2010). In such cases, modulations of sound‐filtering tissues that are peripheral to the larynx (e.g., pharyngeal and orofacial musculature associated with the lips, tongue, nasal cavities and jaws) are employed to adjust sound production to match those of an acoustic model (Matrosova et al., 2007; Riede & Titze, 2008; Stoeger et al., 2012; Vernes et al., 2021; Volodina et al, 2010). Moreover, if the large‐scale differences that characterize the “bark” and “chirp” are a product of sexually dimorphic physiological and anatomical dimensions, then large scale differences in call structure would be reflected in the entire vocal repertoire of this species; in other words, no call type would be similar between male and female vervets. To the contrary, both males and females produce acoustically similar vocalizations such as grunts, snake chutters, eagle rraups, and intergroup wrr vocalizations (Struhsaker, 1967). While it is likely that many of these calls are distinguishable by sex, the developmental trajectories of sex‐specific alarm calls show a distinct decoupling as the monkeys age into adulthood (Price et al., 2015; Struhsaker, 1967), in a manner that is not observed in sexually monomorphic vocalizations such as grunts (Dubreuil, 2019).

An additional dimension to the vervet alarm call story lies in the fact that, in several species (including vervets), calls produced in response to terrestrial predators are acoustically similar to calls produced in other high‐arousal situations, including within/between group aggression, or in contexts associated with competition over access to resources (Fichtel & Kappeler, 2002; Fischer et al., 2002; Fischer, 2017; Price et al., 2015; Wheeler, 2009). Taken together, the evidence suggests that the referential function regarding specific predator threats that has been traditionally ascribed to vervet alarm calls may be peripheral, or incidental to a more generalized alerting function that also conveys information regarding specific attributes of the signaler. In line with this possibility, recent work has highlighted how calling events (in particular the threat alarm bark) among male vervets increase in frequency during the breeding season, and that higher ranking males are more likely to produce barks than lower ranking ones, suggesting that the threat alarm bark may play a role in advertising male quality (Schad et al., 2023). A similar hypothesis regarding the potentially sexually selective function of alarm calls has been proposed for the sexually distinct alarms of the closely related Diana monkeys (*Cercopithecus diana*), which are also characterized by sexual dimorphism at adulthood (Zuberbühler et al., 1997).

Sex differences in the tendency for males and females to produce barks versus chirps represent an instance of signal concordance, where the sex of the caller shows a reasonably consistent relationship with the types of call produced by a given individual (Fuller & Cords, 2017). While the selective pressures underlying these differences remain to be explored more fully, the specific acoustic parameters that distinguish the male bark from the female chirp (i.e., Low DF1, DFA2, and longer call and call element duration) do generate some possible hypotheses. Although not tested here, these variables seem well adapted to act as an index of caller body size vis‐a‐vis other males, and may provide cues relating to a caller's ability, or even their motivation to compete over access to resources (Fitch & Hauser, 2003). Future work should explore the relationship between the acoustic features of male barks and caller age, rank, body size, and mating success to explore this possibility.

An additional question to emerge from this analysis is why, and under what circumstances, the acoustic properties of alarm calls of males and females do converge occasionally, as evidenced by our cluster analysis that showed acoustic overlap between some male and female terrestrial alarm calls. This overlap suggests that some calls are “intermediate” and grade between the two qualitative types. There is as yet no consensus on the mechanistic causes behind graded calls, and what functional significance they may hold for receivers. One possibility is that the graded variation that characterizes the acoustic structure of terrestrial alarm calls mirrors variations in the internal state of different callers within the context of a predator encounter. During a bout of alarm calling, it was our impression that males often moved into trees that were closer to the eliciting predator or predator models. Conversely, females and immature individuals generally maintained their distance, or moved further away (personal observation). These differences in behavior suggest that the sexes may vary in their motivational states during predator encounters, which could be reflected in the type of the alarm call that they produce. This hypothesis would explain why in some instances, individuals produced calls that were more typical of the opposite sex; females may experience higher levels of arousal and different motivational states when they are (for example) closer to the eliciting predator. In line with this hypothesis, Seyfarth & Cheney (1980) also noted that females were more likely to produce alarm calls that were subjectively more similar to those produced by males when they were closer to the predator that elicited the signal. Taken together, these observations suggest that the motivational states of males and females producing these calls may typically differ in contexts that elicit calling, thus affecting the call type each sex tends to produce. Still, motivational states are unlikely to be completely fixed for either sex, and may vary based on (for example) the perceived immediacy of the arousing stimulus (e.g., proximity of a predator or another group).

Another related possibility is that different call types denote different levels of what has been referred to as “potency” in studies focused on the expression of human emotion (Goudbeek & Scherer, 2010). Potency (also referred to as Dominance; Russell, 1994) refers to an individual's sense of control or coping potential over a situation (Goudbeek & Scherer, 2010). In humans, this emotional dimension is said to be high when an individual is experiencing anger, interest, excitement, irritation, or wanting to take action, and low when an individual experiences calmness, apathy, fear, or anxiety (Fontaine et al., 2007; Russell, 1994). High levels of potency are associated with particular vocal cues, feelings, and action tendencies, including an increased volume and assertiveness in the voice, a desire to take action and initiative, and a want to be seen (Fontaine et al., 2007). From the perspective of the vervet monkeys, signals associated with high emotional potency may be associated with aggression towards a predator, or towards male conspecifics. More generally, calls that convey a high level of potency may benefit callers by eliciting evasive responses from receivers. These motivational explanations would need to be tested, perhaps by experimentally altering the size of predator models, or the distance between mounts and callers (see Manser, 2001; Warkentin et al., 2001), in an attempt to determine whether the caller's assessment of a given situation affects whether they produce chirp‐ or bark‐like alarms.

Our study highlights the importance of considering multiple dimensions when classifying acoustic signals into “types”. While the focus on the referential quality of vervet alarm calls has generated ample research and debate over the last 50 years, many accounts of these alarms give no, or only passing mention to sex differences in the structure of these calls. This omission is in many ways surprising, as the acoustic structure of vocal signals is expected to be tied to their function; in particular, how the qualities of sounds affect receiver's directly, how characteristics embedded in the sounds can reveal information about the caller (e.g., body size, identity, exhaustion), or how receiver behavior is affected through learned responses (Owren & Rendall, 2001). We hope that by concentrating on the structure of these alarm calls, and in particular sex differences in call structure, our work will contribute towards a more holistic view of these signals, their function among males and females, and the selective pressures that underlie these structural differences.

Our analysis yields the specific acoustic properties that differentiate the terrestrial alarm calls of male and female vervet monkeys, and in so doing we provide quantitative support for earlier qualitative descriptions of sex differences in these calls. We propose that these sex differences may reflect different selective pressures on males and females that have led to divergence in the acoustic structure in alarms between sexes. For example, in line with other recent research (Schad et al., 2023) the threat alarm bark may function in a manner similar to “loud” or “long” calls produced by males of other species. Future research could probe this possibility further by looking at whether variation in the structure of alarm calls corresponds with a caller's dominance status, number of copulations attained over the breading season, or offspring sired by individual callers.

## AUTHOR CONTRIBUTIONS

**Colin Dubreuil**: Conceptualization (lead); data curation (lead); formal analysis (equal); funding acquisition (lead); investigation (lead); methodology (lead); project administration (equal); writing—original draft (lead); writing—review and editing (equal). **Hugh Notman:** Conceptualization (equal); methodology (equal); project administration (equal); resources (equal); supervision (lead); writing—original draft (equal); writing—review and editing (equal). **Louise Barrett**: Conceptualization (supporting); project administration (equal); resources (equal). **Peter Henzi**: Conceptualization (equal); data curation (equal); formal analysis (equal); methodology (supporting); project administration (equal); resources (equal); supervision (supporting); visualization (equal); writing—original draft (equal); writing—review and editing (equal). **Mary Susan McDonald Pavelka:** Conceptualization (supporting); project administration (equal); resources (equal).

## CONFLICT OF INTEREST STATEMENT

The authors declare no conflicts of interest.

## Supporting information

Supporting information.

Supporting information.

## Data Availability

Our data will be made available through an online repository, pending the acceptance of this article.
