# Supplementary material for: Sex differences in the acoustic structure of terrestrial alarm calls in vervet monkeys (Chlorocebus pygerythrus)
Source: Am J Primatol. 2024 Aug 20;87(1):e23674. doi: 10.1002/ajp.23674 (PMC11650924; doi:10.1002/ajp.23674)
Supplement: Supplementary file 1 — Supporting information. [file AJP-87-e23674-s002.docx]

**Supplementary Information 1 (SI1)**

**Results of Random forest model using SMOTE algorithm**

The second random forest model (using the SMOTE algorithm for unbalanced samples) was able to distinguish between the calls of males and females with a high rate of accuracy (95.29% accuracy, OOB error = 2.07%, sensitivity = 98.39%, specificity = 95.65%). Classification error was low for calls produced by both males, and females (Males = 0.03, Females = 0.01). The model accurately predicted the caller’s sex 97.65% of the time from the validation data set (95% CIs: 91.76, 99.71), which was significantly better than chance expectation (permutation test; p < 0.001, expected classification accuracy by chance = ~61%). Calls produced by both males and females were classified correctly at levels that exceeded chance (Adult males: 98.39 % accuracy vs ~73% accuracy expected by chance, p < 0.001; Adult females: 95.65 % accuracy vs ~27 % accuracy expected by chance, p < 0.001).

Variable importance measures (mean decrease in Gini and Accuracy) differed somewhat in terms of their ranking of the eight representative acoustic variables (Table A1). Still, both Gini and model accuracy indexes rated DF1 as the most important variable for distinguishing between the calls of males and females. As was the case for our original model (see main text), DFA2 and Average element length were rated as the second and third most important variables in the classification task, though Average element length was rated as slightly more important than DFA2 when considering decrease in accuracy. Parameters related to the relative position of the minimum and maximum peak frequency (pfmiloc, pfmaxloc), call duration, and the movement of the peak frequency (pfjump) were rated as relatively low in terms of importance for discriminating between the sexes, though not necessarily in the same order as they were for our first model (see table 1A for comparison of variable importance between models). As was the case in our first model (see main text) the number of elements in the call was rated as the least important variable for distinguishing between the calls of males and females.

Table a1: variable importance (mean decrease in Accuracy and Gini) for the balanced random forest model.(balanced using the SMOTE algorithm) as well as the unbalanced model discussed in the main text. Importance measures are scaled to have a maximum value of 100 for ease of comparison.

|  | Unbalanced Model | | | SMOTE balanced model | |
| --- | --- | --- | --- | --- | --- |
| Acoustic Variable | Decrease in Accuracy | Decrease in Gini | Decrease in Accuracy | | Decrease in  Gini |
| DF1 | 100.00 | 100.00 | 100.00 | | 100.00 |
| DFA2 | 57.22 | 75.15 | 22.80 | | 39.84 |
| Mean element length | 46.19 | 35.54 | 36.94 | | 13.46 |
| Call Duration | 7.27 | 11.76 | 7.73 | | 1.15 |
| pfmaloc | 5.97 | 10.38 | 3.70 | | 2.87 |
| pfjump | 1.77 | 7.67 | 5.03 | | 0.57 |
| pfmiloc | 1.61 | 3.09 | 5.41 | | 0.56 |
| Number of elements | 0.00 | 0.00 | 0.00 | | 0.00 |
