# Supplementary material for: Sex differences in the acoustic structure of terrestrial alarm calls in vervet monkeys (Chlorocebus pygerythrus)
Source: Am J Primatol. 2024 Aug 20;87(1):e23674. doi: 10.1002/ajp.23674 (PMC11650924; doi:10.1002/ajp.23674)
Supplement: Supplementary file 2 — Supporting information. [file AJP-87-e23674-s001.docx]

**Supplementary Information 2 (SI2)**

**Sex differences in the representative acoustic variables: Full model results**

Table S1. Model estimates (standardized beta coefficients) of the effects of caller sex (fixed effect) and caller identity (random effect) on DF1. Model family = gamma. Links - mu = log; shape = identity. Number of observations: 286.

|  | **Estimate** | | **Est. Error** | **L-95% CI** | **U-95% CI** | **Rhat** | **Bulk_ESS** | **Tail_ESS** | **Pd** |
| --- | --- | --- | --- | --- | --- | --- | --- | --- | --- |
| **Population level effects** | | | | | | | | | |
| Intercept | 7.13 | | 0.15 | 6.84 | 7.42 | 1.01 | 981 | 1434 | 100% |
| Sex Male | -1.28 | | 0.18 | -1.64 | -0.94 | 1.01 | 877 | 1097 | 100% |
| **Group level effects**  **Caller (number of levels: 27)** | | | | | | | | | |
| sd(Intercept) | 0.40 | | 0.07 | 0.29 | 0.56 | 1.01 | 925 | 1297 |  |
| **Family Specific Parameters** | | | | | | | | | |
| Shape | 22.17 | | 1.93 | 18.67 | 26.02 | 1.00 | 3436 | 3052 |  |
| **Full model R2 values** | | **Estimate** | | **Estimated error** | | **Q2.5** | | **Q97.5** | |
| R^2^ conditional | | 0. 9023665 | | 0. 008242952 | | 0. 8826773 | | 0. 914879 | |
| R^2^ Marginal | | 0. 5517967 | | 0. 1309274 | | 0. 2680435 | | 0. 7662134 | |

For each parameter, Rhat indicates whether or not the model has converged (at convergence, Rhat ≈ 1). Bulk_ESS and Tail_ESS are measures of effective sample size. R^2^ values are included as an indication of the variance explained by the model.

Table S2. Model estimates (standardized beta coefficients) of the effects of caller sex (fixed effect) and caller identity (random effect) on DFA2. Model family = gamma. Links - mu = log; shape = identity. Number of observations: 286.

|  | **Estimate** | | **Est. Error** | **L-95% CI** | **U-95% CI** | **Rhat** | **Bulk_ESS** | **Tail_ESS** | **pd** |
| --- | --- | --- | --- | --- | --- | --- | --- | --- | --- |
| **Population level effects** | | | | | | | | | |
| Intercept | 7.89 | | 0.09 | 7.71 | 8.06 | 1.00 | 1960 | 2671 | 100% |
| Sex Male | -0.29 | | 0.11 | -0.51 | -0.08 | 1.00 | 1521 | 2440 | 99.35% |
| **Group level effects**  **Caller (number of levels: 27)** | | | | | | | | | |
| sd(Intercept) | 0.25 | | 0.05 | 0.18 | 0.35 | 1.00 | 1631 | 2521 |  |
| **Family Specific Parameters** | | | | | | | | | |
| Shape | 33.04 | | 2.94 | 27.59 | 38.97 | 1.00 | 5631 | 5294 |  |
| **Full model R2 values** | | **Estimate** | | **Estimated error** | | **Q2.5** | | **Q97.5** | |
| R^2^ conditional | | 0.7245944 | | 0.02227317 | | 0.674501 | | 0.7619631 | |
| R^2^ Marginal | | 0.2639038 | | 0.1399212 | | 0.01671203 | | 0.5315933 | |

For each parameter, Rhat indicates whether or not the model has converged (at convergence, Rhat ≈ 1). Bulk_ESS and Tail_ESS are measures of effective sample size. R^2^ values are included as an indication of the variance explained by the model.

Table S3. Model estimates (standardized beta coefficients) of the effects of caller sex (fixed effect) and caller identity (random effect) on pfjump. Model family = gamma. Links - mu = log; shape = identity. Number of observations: 286.

|  | **Estimate** | | **Est. Error** | **L-95% CI** | **U-95% CI** | **Rhat** | **Bulk_ESS** | **Tail_ESS** | **pd** |
| --- | --- | --- | --- | --- | --- | --- | --- | --- | --- |
| **Population level effects** | | | | | | | | | |
| Intercept | 6.33 | | 0.18 | 5.98 | 6.68 | 1.00 | 5168 | 5097 | 100% |
| Sex Male | 0.33 | | 0.21 | -0.10 | 0.74 | 1.00 | 4303 | 5114 | 93.40% |
| **Group level effects**  **Caller (number of levels: 27)** | | | | | | | | | |
| sd(Intercept) | 0.40 | | 0.09 | 0.25 | 0.61 | 1.00 | 3063 | 4256 |  |
| **Family Specific parameters:** | | | | | | | | | |
| Shape | 1.49 | | 0.12 | 1.27 | 1.74 | 1.00 | 5810 | 5810 |  |
| **Full model R2 values** | | **Estimate** | | **Estimated error** | | **Q2.5** | | **Q97.5** | |
| R^2^ conditional | | 0. 185515 | | 0. 05110134 | | 0. 09843266 | | 0. 2965849 | |
| R^2^ Marginal | | 0. 0187991 | | 0. 0178613 | | 7.695835e-05 | | 0. 06467793 | |

For each parameter, Rhat indicates whether or not the model has converged (at convergence, Rhat ≈ 1). Bulk_ESS and Tail_ESS are measures of effective sample size. R^2^ values are included as an indication of the variance explained by the model.

Table S4. Model estimates (standardized beta coefficients) of the effects of caller sex (fixed effect) and caller identity (random effect) on pfmaloc. Model family = gamma. Links - mu = log; shape = identity. Number of observations: 286.

|  | **Estimate** | | **Est. Error** | **L-95% CI** | **U-95% CI** | **Rhat** | **Bulk_ESS** | **Tail_ESS** | **pd** |
| --- | --- | --- | --- | --- | --- | --- | --- | --- | --- |
| **Population level effects** | | | | | | | | | |
| Intercept | -1.38 | | 0.20 | -1.77 | -1.00 | 1.00 | 4873 | 4952 | 100% |
| Sex Male | -0.64 | | 0.23 | -1.11 | -0.17 | 1.00 | 5155 | 5131 | 99.67% |
| **Group level effects**  **Caller (number of levels: 27** | | | | | | | | | |
| sd(Intercept) | 0.42 | | 0.12 | 0.21 | 0.69 | 1.00 | 2280 | 3816 |  |
| **Family Specific parameters:** | | | | | | | | | |
| Shape | 1.08 | | 0.08 | 0.93 | 1.25 | 1.00 | 10175 | 5385 |  |
| **Full model R2 values** | | **Estimate** | | **Estimated error** | | **Q2.5** | | **Q97.5** | |
| R^2^ conditional | | 0. 124819 | | 0. 0415299 | | 0. 05619125 | | 0. 2197881 | |
| R^2^ Marginal | | 0. 06224066 | | 0. 04582406 | | 0. 002932942 | | 0. 1781208 | |

For each parameter, Rhat indicates whether or not the model has converged (at convergence, Rhat ≈ 1). Bulk_ESS and Tail_ESS are measures of effective sample size. R^2^ values are included as an indication of the variance explained by the model.

Table S5. Model estimates (standardized beta coefficients) of the effects of caller sex (fixed effect) and caller identity (random effect) on pfmiloc. Model family = skew_normal. Links - mu = identity; sigma = identity; alpha = identity. Number of observations: 286.

|  | **Estimate** | | **Est. Error** | **L-95% CI** | **U-95% CI** | **Rhat** | **Bulk_ESS** | **Tail_ESS** | **pd** |
| --- | --- | --- | --- | --- | --- | --- | --- | --- | --- |
| **Population level effects** | | | | | | | | | |
| Intercept | 0.69 | | 0.02 | 0.65 | 0.72 | 1.00 | 8086 | 6472 | 100% |
| Sex Male | 0.01 | | 0.02 | -0.02 | 0.04 | 1.00 | 9204 | 5203 | 74.44% |
| **Group level effects**  **Caller (number of levels: 27)** | | | | | | | | | |
| sd(Intercept) | 0.01 | | 0.01 | 0.00 | 0.03 | 1.00 | 4419 | 4085 |  |
| **Family Specific parameters:** | | | | | | | | | |
| Sigma | 0.25 | | 0.01 | 0.23 | 0.27 | 1.00 | 7296 | 6349 |  |
| Alpha | -14.32 | | 2.50 | -19.51 | -9.77 | 1.00 | 11727 | 5923 |  |
| **Full model R2 values** | | **Estimate** | | **Estimated error** | | **Q2.5** | | **Q97.5** | |
| R^2^ conditional | | 0. 002215354 | | 0. 002711131 | | 4.231211e-05 | | 0. 009699293 | |
| R^2^ Marginal | | 0. 001022822 | | 0. 001530606 | | 1.143196e-06 | | 0. 005196329 | |

For each parameter, Rhat indicates whether or not the model has converged (at convergence, Rhat ≈ 1). Bulk_ESS and Tail_ESS are measures of effective sample size. R^2^ values are included as an indication of the variance explained by the model.

Table S6. Model estimates (standardized beta coefficients) of the effects of caller sex (fixed effect) and caller identity (random effect) on Average Element Length. Model family = student. Links - mu = identity; sigma = identity; nu = identity. Number of observations: 286.

|  | **Estimate** | | **Est. Error** | **L-95% CI** | **U-95% CI** | **Rhat** | **Bulk_ESS** | **Tail_ESS** | **pd** |
| --- | --- | --- | --- | --- | --- | --- | --- | --- | --- |
| **Population level effects** | | | | | | | | | |
| Intercept | 0.06 | | 0.01 | 0.05 | 0.07 | 1.00 | 3389 | 4856 | 100% |
| Sex Male | 0.03 | | 0.01 | 0.02 | 0.05 | 1.00 | 3091 | 4824 | 100% |
| **Group level effects**  **Caller (number of levels: 27)** | | | | | | | | | |
| sd(Intercept) | 0.01 | | 0.00 | 0.01 | 0.02 | 1.00 | 2312 | 3660 |  |
| **Family Specific parameters:** | | | | | | | | | |
| Sigma | 0.02 | | 0.00 | 0.02 | 0.02 | 1.00 | 9033 | 5523 |  |
| Nu | 26.84 | | 14.11 | 8.58 | 62.06 | 1.00 | 9704 | 6014 |  |
| **Full model R2 values** | | **Estimate** | | **Estimated error** | | **Q2.5** | | **Q97.5** | |
| R^2^ conditional | | 0. 4433369 | | 0. 03446405 | | 0. 371127 | | 0. 5044935 | |
| R^2^ Marginal | | 0. 2886487 | | 0. 07436395 | | 0. 131739 | | 0. 4220669 | |

For each parameter, Rhat indicates whether or not the model has converged (at convergence, Rhat ≈ 1). Bulk_ESS and Tail_ESS are measures of effective sample size. R^2^ values are included as an indication of the variance explained by the model.

Table S7. Model estimates (standardized beta coefficients) of the effects of caller sex (fixed effect) and caller identity (random effect) on Call Duration. Model family = gamma. Links: mu = log; shape = identity. Number of observations: 286.

|  | **Estimate** | | **Est. Error** | **L-95% CI** | **U-95% CI** | **Rhat** | **Bulk_ESS** | **Tail_ESS** | **pd** |
| --- | --- | --- | --- | --- | --- | --- | --- | --- | --- |
| **Population level effects** | | | | | | | | | |
| Intercept | -1.85 | | 0.11 | -2.08 | -1.63 | 1.00 | 5649 | 5238 | 100% |
| Sex Male | 0.50 | | 0.13 | 0.23 | 0.76 | 1.00 | 5239 | 5099 | 99.94% |
| **Group level effects**  **Caller (number of levels: 27** | | | | | | | | | |
| sd(Intercept) | 0.23 | | 0.06 | 0.13 | 0.36 | 1.00 | 2989 | 4296 |  |
| **Family Specific parameters:** | | | | | | | | | |
| Shape | 3.15 | | 0.26 | 2.66 | 3.67 | 1.00 | 9997 | 5959 |  |
| **Full model R2 values** | | **Estimate** | | **Estimated error** | | **Q2.5** | | **Q97.5** | |
| R^2^ conditional | | 0. 1527855 | | 0. 03610922 | | 0. 08799458 | | 0. 2288073 | |
| R^2^ Marginal | | 0. 0758473 | | 0. 03322329 | | 0. 01787354 | | 0. 1461379 | |

For each parameter, Rhat indicates whether or not the model has converged (at convergence, Rhat ≈ 1). Bulk_ESS and Tail_ESS are measures of effective sample size. R^2^ values are included as an indication of the variance explained by the model.

Table S8. Model estimates (standardized beta coefficients) of the effects of caller sex (fixed effect) and caller identity (random effect) on number of elements. Model family = poisson. Links: mu = log. Offset = call duration. Number of observations: 286.

|  | **Estimate** | **Est. Error** | **L-95% CI** | **U-95% CI** | **Rhat** | **Bulk_ESS** | **Tail_ESS** | **pd** |
| --- | --- | --- | --- | --- | --- | --- | --- | --- |
| **Population level effects** | | | | | | | | |
| Intercept | 2.67 | 0.08 | 2.51 | 2.82 | 1.00 | 8785 | 5482 | 100% |
| Sex Male | -0.41 | 0.09 | -0.59 | -0.23 | 1.00 | 8823 | 5429 | 100% |
| **Group level effects**  **Caller (number of levels: 27)** | | | | | | | | |
| sd(Intercept) | 0.05 | 0.04 | 0.00 | 0.14 | 1.00 | 4638 | 4738 |  |
| **Full model R2 values** | **Estimate** | | **Estimated error** | | **Q2.5** | | **Q97.5** | |
| R^2^ conditional | 0. 8498357 | | 0. 007358295 | | 0. 8326647 | | 0. 8626276 | |
| R^2^ Marginal | 0. 8499076 | | 0. 005208916 | | 0. 8360587 | | 0. 8556195 | |

For each parameter, Rhat indicates whether or not the model has converged (at convergence, Rhat ≈ 1). Bulk_ESS and Tail_ESS are measures of effective sample size. R^2^ values are included as an indication of the variance explained by the model.

**Sex differences in cluster membership: Full model results and individual contributions to clusters**

Table S9. Model estimates (standardized beta coefficients) of the effects of caller sex (fixed effect) and caller identity (random effect) on cluster membership. Reference values: Cluster 2, Males. Model was run within a Bayesian framework, using four chains and 3000 iterations. Model family = categorical. mu2 = logit, mu3 = logit. Number of observations: 286.

|  | **Estimate** | | **Est. Error** | | **L-95% CI** | | **U-95% CI** | | **Rhat** | **Bulk_ESS** | | **Tail_ESS** | **pd** |
| --- | --- | --- | --- | --- | --- | --- | --- | --- | --- | --- | --- | --- | --- |
| **Population level effects** | | | | | | | | | | | | | |
| mu 1 – Intercept | 2.40 | | 0.60 | | 1.13 | | 3.57 | | 1.00 | 3918 | | 4581 | 99.9% |
| mu 3 – Intercept | -4.83 | | 2.00 | | -9.70 | | -1.89 | | 1.00 | 4407 | | 3174 | 100% |
| mu 1 – Sex Female | -3.31 | | 1.00 | | -4.95 | | -1.06 | | 1.00 | 2178 | | 4190 | 99.68% |
| mu 3 – Sex Female | -0.49 | | 0.94 | | -2.29 | | 1.35 | | 1.00 | 10413 | | 6315 | 70.43% |
| **Group level effects**  **Caller (number of levels: 27)** | | | | | | | | | | | | | |
| sd(mu 1 – Intercept) | | 2.02 | | 1.11 | | 0.55 | | 4.48 | 1.01 | | 1457 | 2747 |  |
| sd(mu 3 – Intercept) | | 2.87 | | 1.24 | | 1.18 | | 5.95 | 1.00 | | 4601 | 3740 |  |

For each parameter, Rhat indicates whether or not the model has converged (at convergence, Rhat ≈ 1). Bulk_ESS and Tail_ESS are measures of effective sample size. Note that the categorical family can’t provide R^2^ values.

Table S10: Cluster assignment for calls produced by all individuals in the analysis. Numbers in brackets represent the percentage of each individual’s calls that contributed to each of the three clusters. Grayed out cells represent cases where a given individual contributed no calls to a given cluster.

| Caller Sex | Caller ID | No. calls in analysis | No. calls in Cluster 1 | No. calls in Cluster 2 | No. calls in Cluster 3 |
| --- | --- | --- | --- | --- | --- |
| Male | Alla | 7 | 7 (100%) | 0 (0%) | 0 (0%) |
|  | Bone | 20 | 20 (100%) | 0 (0%) | 0 (0%) |
|  | Egon | 29 | 28 (96.6%) | 1 (3.4%) | 0 (0%) |
|  | Flyn | 21 | 17 (81%) | 4 (19%) | 0 (0%) |
|  | Hunt | 31 | 16 (51.6%) | 4 (12.9%) | 11 (35.5%) |
|  | Keit | 5 | 5 (100%) | 0 (0%) | 0 (0%) |
|  | Macy | 3 | 3 (100%) | 0 (0%) | 0 (0%) |
|  | Magn | 5 | 4 (80%) | 1 (20%) | 0 (0%) |
|  | Mori | 21 | 21 (100%) | 0 (0%) | 0 (0%) |
|  | Nige | 8 | 6 (75%) | 2 (25%) | 0 (0%) |
|  | Obi | 3 | 3 (100%) | 0 (0%) | 0 (0%) |
|  | Panc | 6 | 6 (100%) | 0 (0%) | 0 (0%) |
|  | Razo | 7 | 6 (85.7%) | 1 (14.3%) | 0 (0%) |
|  | Schm | 16 | 16 (100%) | 0 (0%) | 0 (0%) |
|  | Wall | 8 | 8 (100%) | 0 (0%) | 0 (0%) |
|  | Yoda | 6 | 6 (100%) | 0 (0%) | 0 (0%) |
|  | Zool | 11 | 11 (100%) | 0 (0%) | 0 (0%) |
|  | Total Male | 207 | 183 (88.4%) | 13 (6.3%) | 11 (5.3%) |
| Female | Arwe | 5 | 2 (40%) | 3 (60%) | 0 (0%) |
|  | Cycl | 1 | 0 (0%) | 1 (100%) | 0 (0%) |
|  | Herm | 12 | 1 (8.3%) | 11 (91.7%) | 0 (0%) |
|  | Holl | 16 | 2 (12.5%) | 14 (87.5%) | 0 (0%) |
|  | Phoe | 19 | 0 (0%) | 19 (100%) | 0 (0%) |
|  | Pris | 18 | 0 (0%) | 17 (94.4%) | 1 (5.6%) |
|  | Sash | 6 | 0 (0%) | 6 (100%) | 0 (0%) |
|  | Turt | 1 | 0 (0%) | 1 (100%) | 0 (0%) |
|  | Tyva | 1 | 0 (0%) | 1 (100%) | 0 (0%) |
|  | Total Female | 79 | 5 (6.3%) | 73 (92.4%) | 1 (1.3%) |
